# Supplementary material for: Mother-child autonomic nervous system interaction as an indication of parental stress: 24-hour cross recurrence plot analysis
Source: PLoS One. 2025 Sep 12;20(9):e0320175. doi: 10.1371/journal.pone.0320175 (PMC12431278; doi:10.1371/journal.pone.0320175)
Supplement: S1 File — This file contains a table listing the abbreviations used in the paper and their meanings, as well as the complete set of recurrence plots and cross-recurrence plots generated for the analyses reported in the paper. (PDF) [file pone.0320175.s001.pdf]

Appendix

S1 Table. Abbreviations and Their Meaning.

**Abbreviations**	**Meanings**
ANS	Autonomic Nervous System
HRV	Heart Rate Variability
RP	Recurrence Plot
CRP	Cross Recurrent Plot
ECG	Electrocardiogram
PSI	Parenting Stress Index
SDNN	Standard Deviation of N-N Intervals
CSI	Cardiac Sympathetic Index
CVI	Cardiac Vagal Index
R-R interval	R peak to R peak intervals of the heartbeat data
N-N interval	Peak-peak intervals extracted from R-R intervals by filtering the artifacts and noise from the heartbeat data
RMSE	Root Mean Squared Error = $\sqrt{\frac{one1}{n}\sum_{i=1}^{n}(y_i - \hat{y}_i)^2}$
MAE	Mean Absolute Error = $\frac{1}{n}\sum_{i=1}^{n}\lvert y_i - \hat{y}_i\rvert$

S2 Figure. The CRPs and SOM Results.

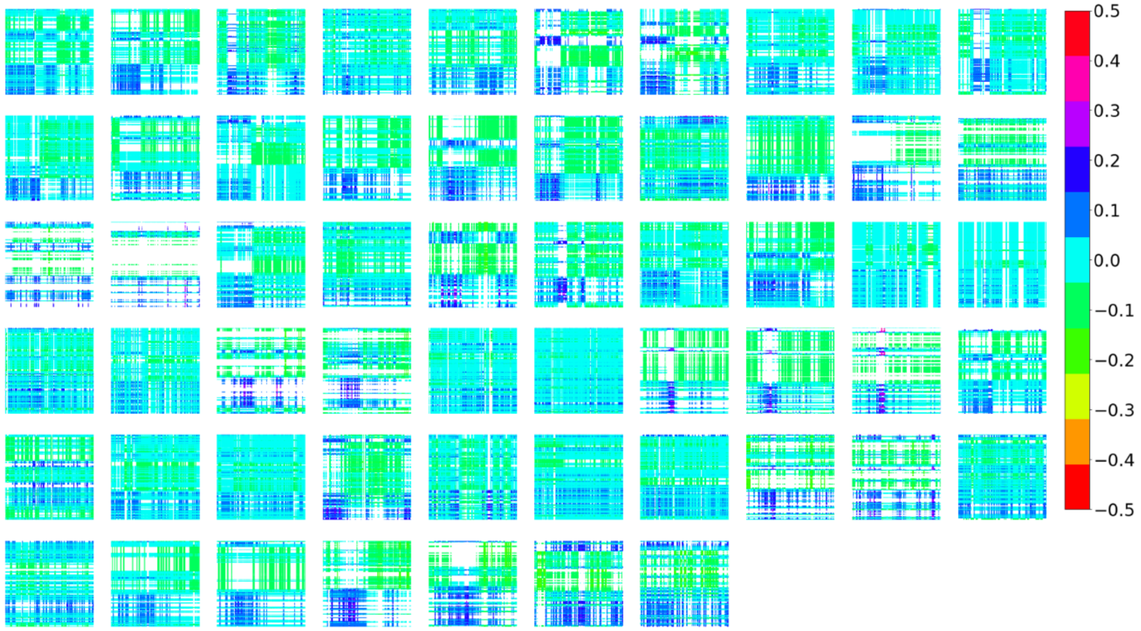

A. SDNN CRPs

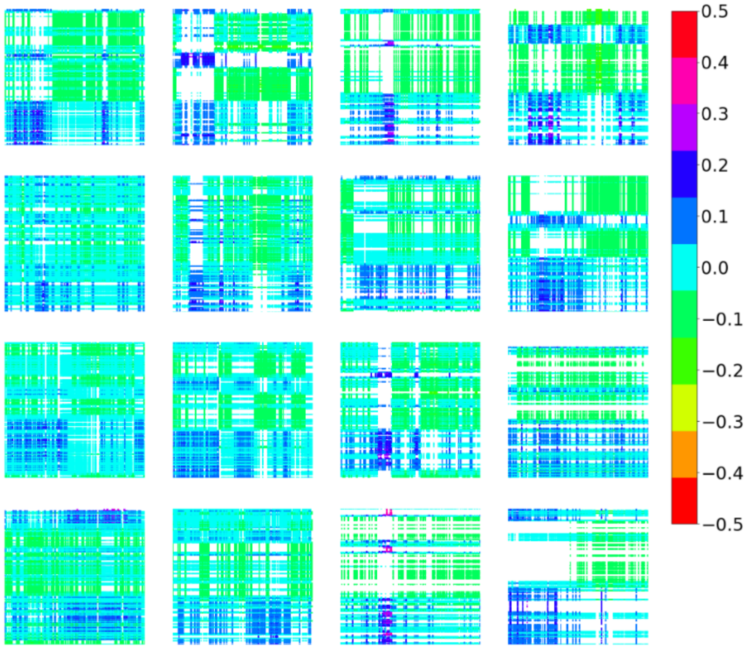

B. SDNN CRPs SOM results

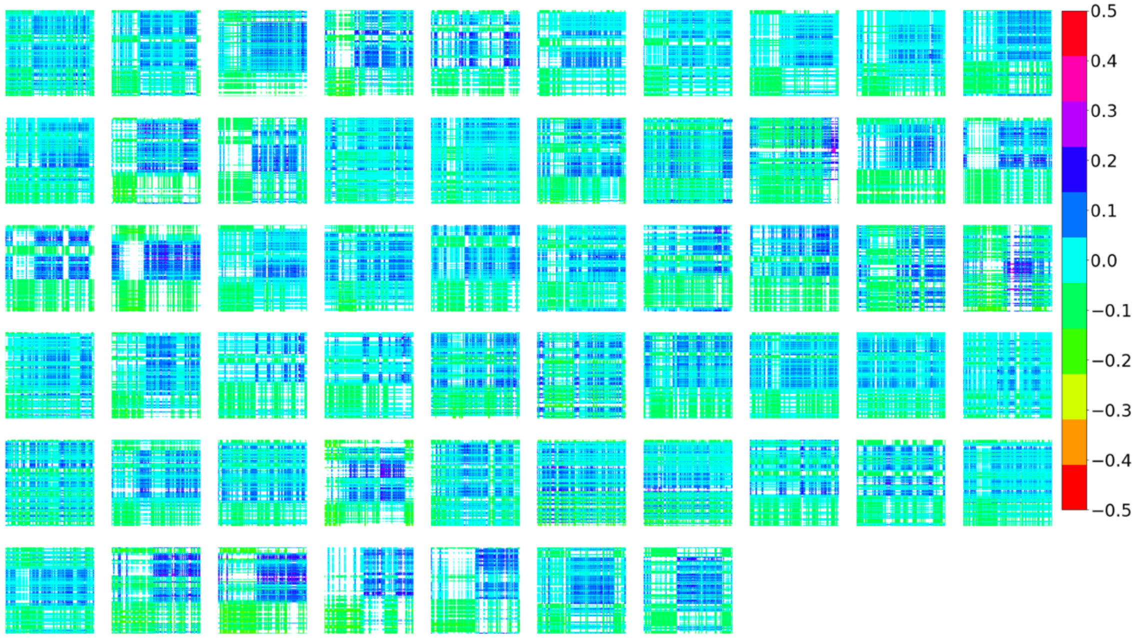

C. CSI CRPs

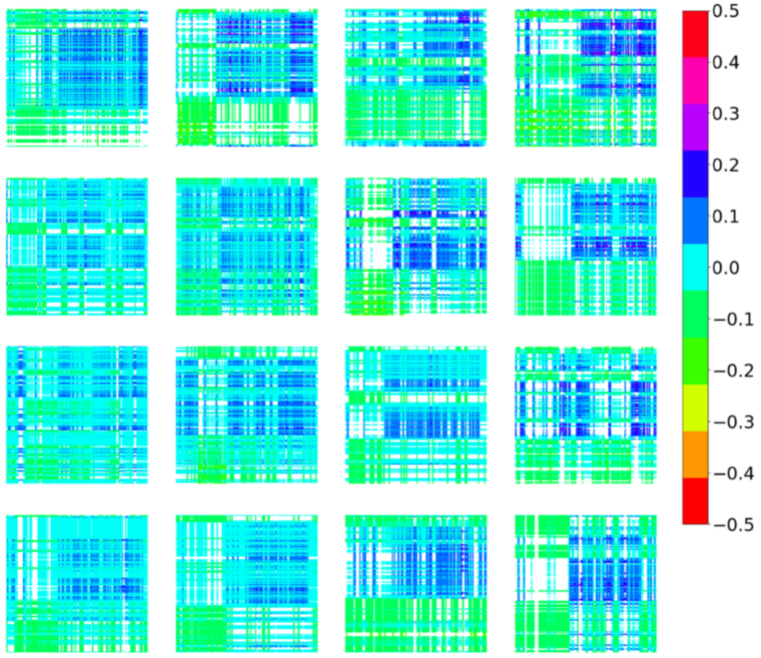

D. CSI CRPs SOM results

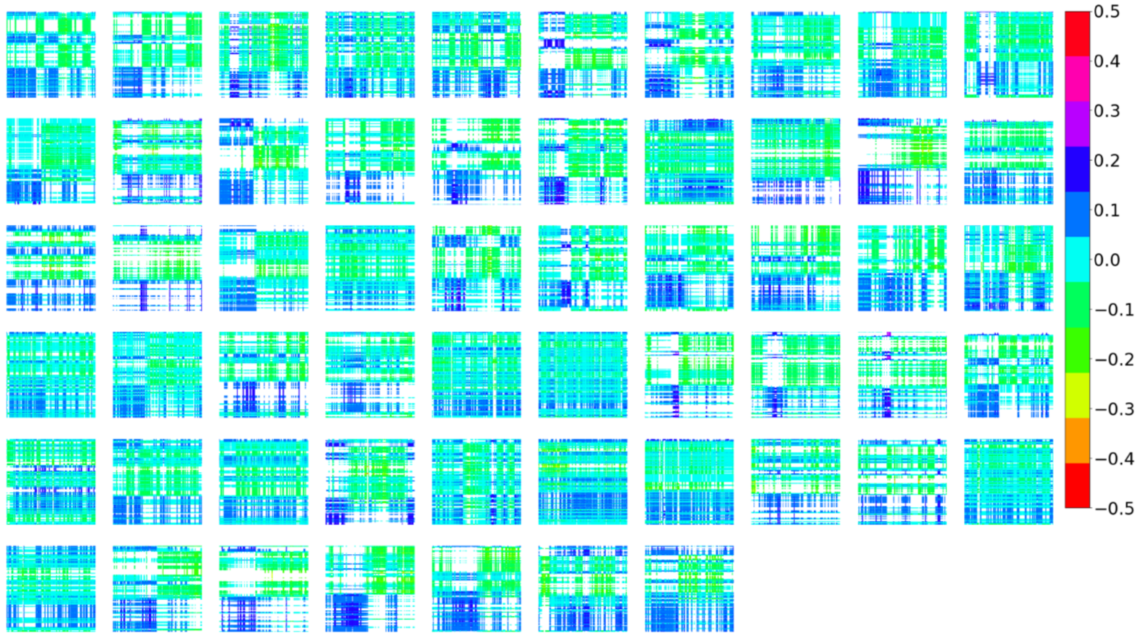

E. CVI CRPs

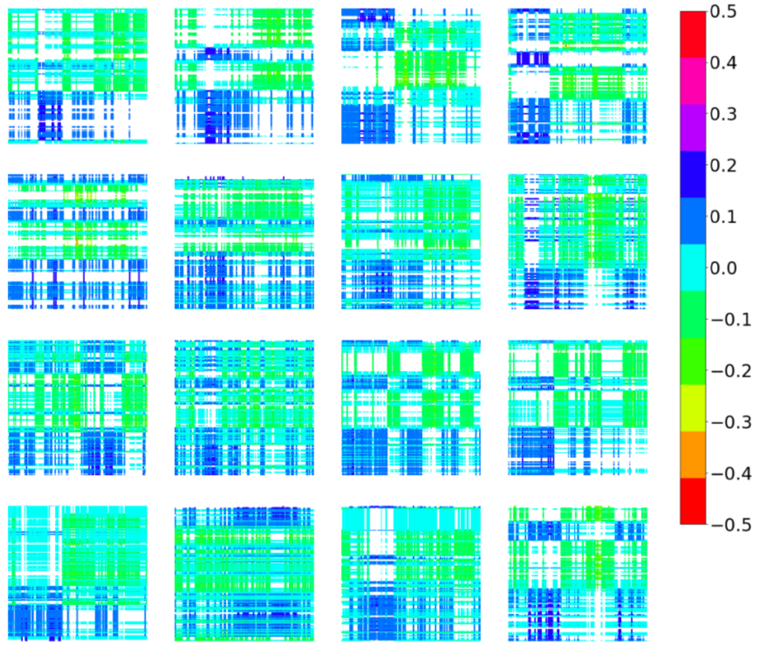

F. CVI CRPs SOM results

S3 Figure. The RPs and SOM Results.

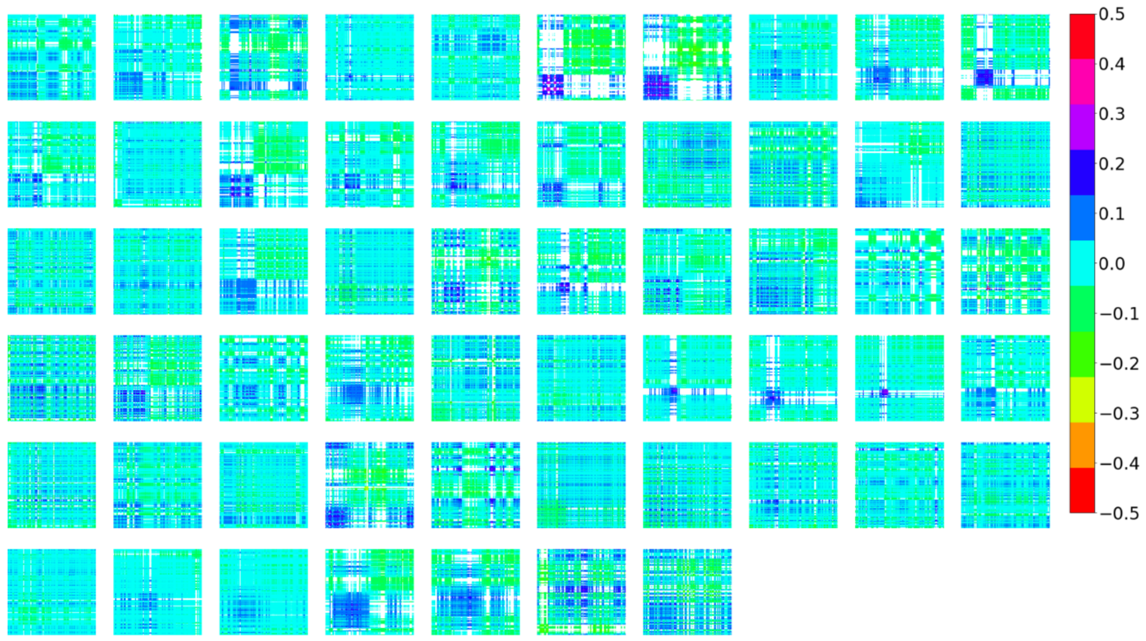

A. Mothers' SDNN

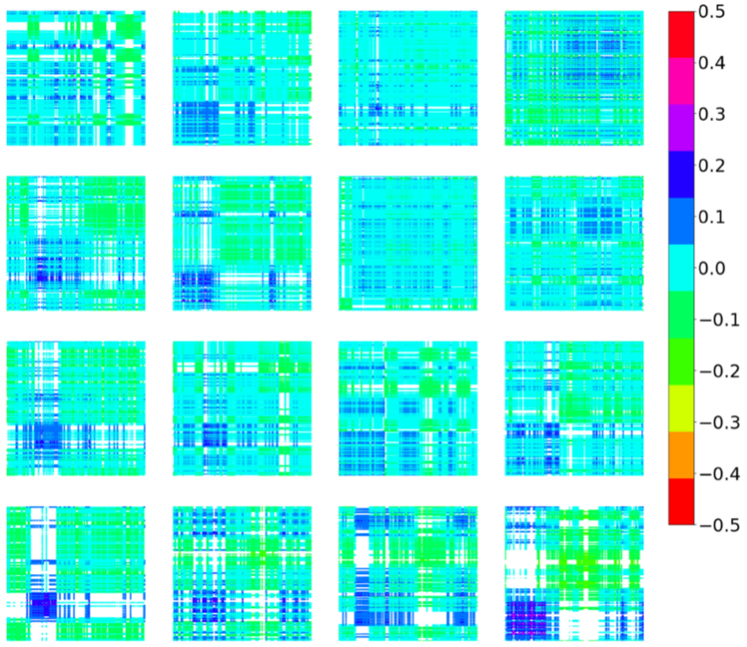

B. Mothers' SDNN RPs SOM

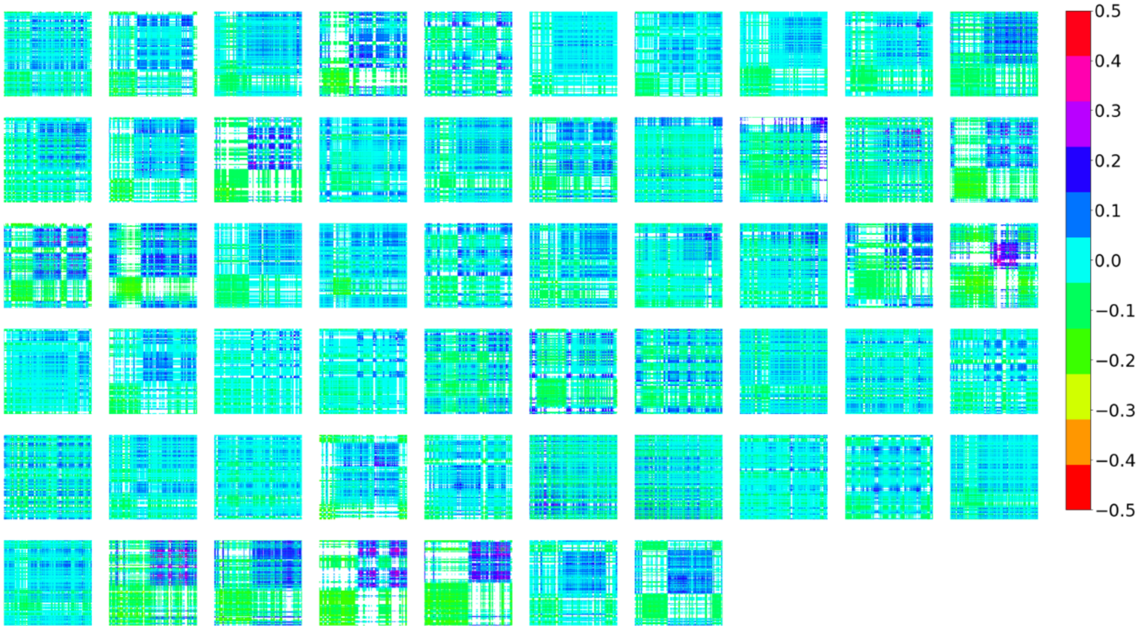

C. Mothers' CSI

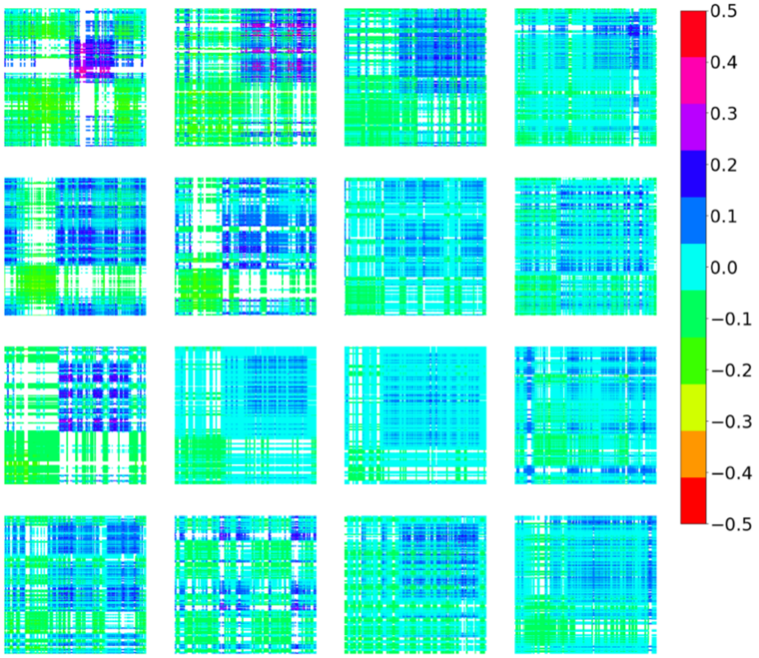

D. Mothers' CSI RPs SOM

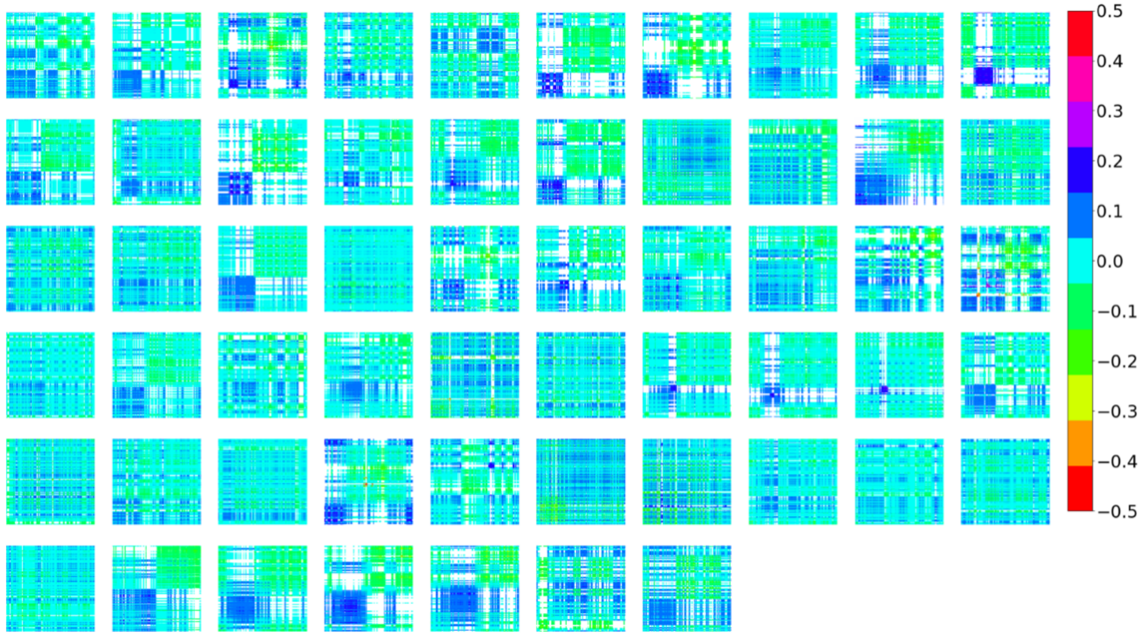

E. Mothers’ CVI

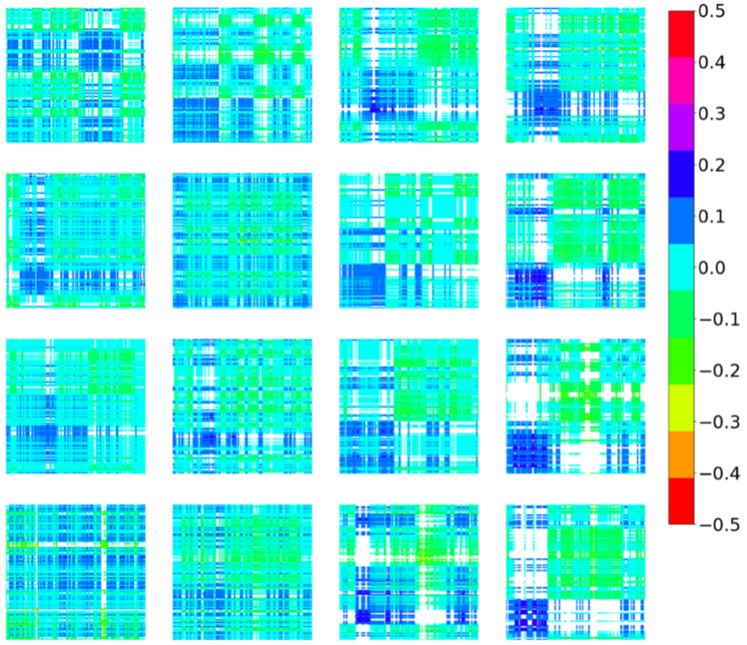

F. Mothers’ CVI RPs SOM

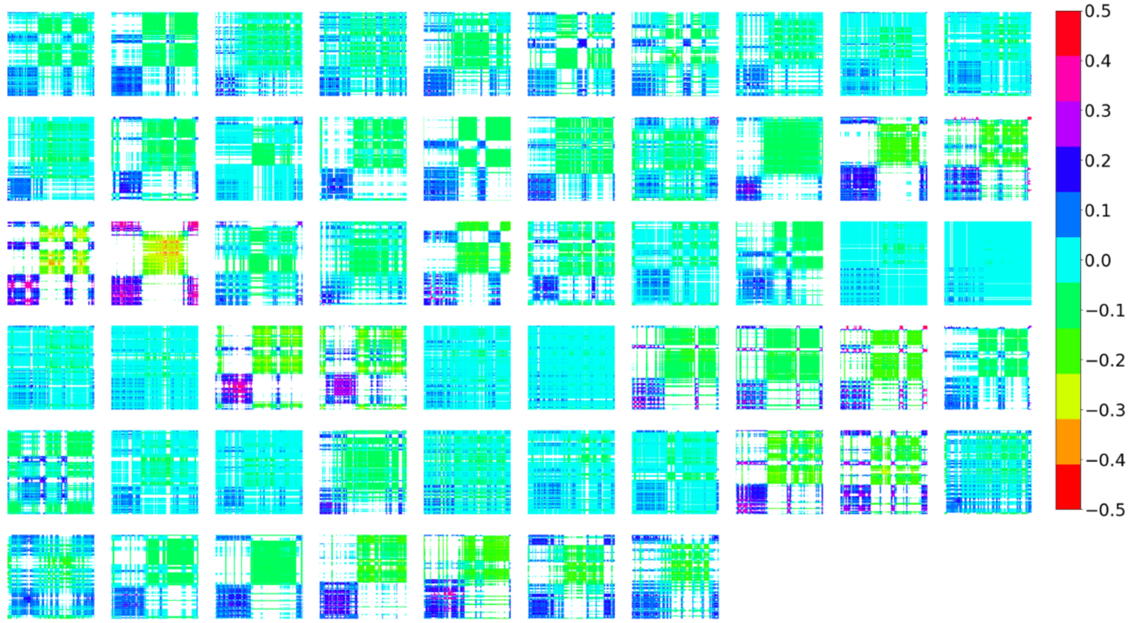

G. Children's SDNN

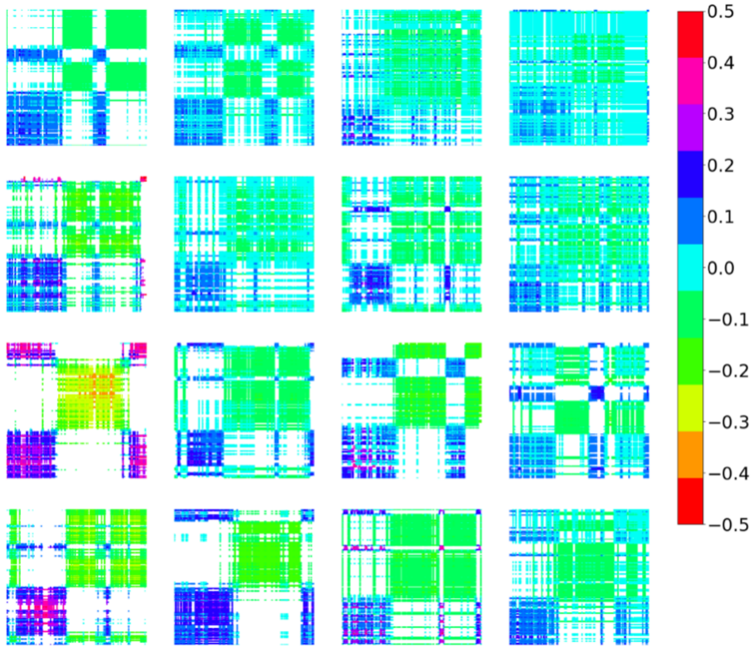

H. Children's SDNN RPs SOM

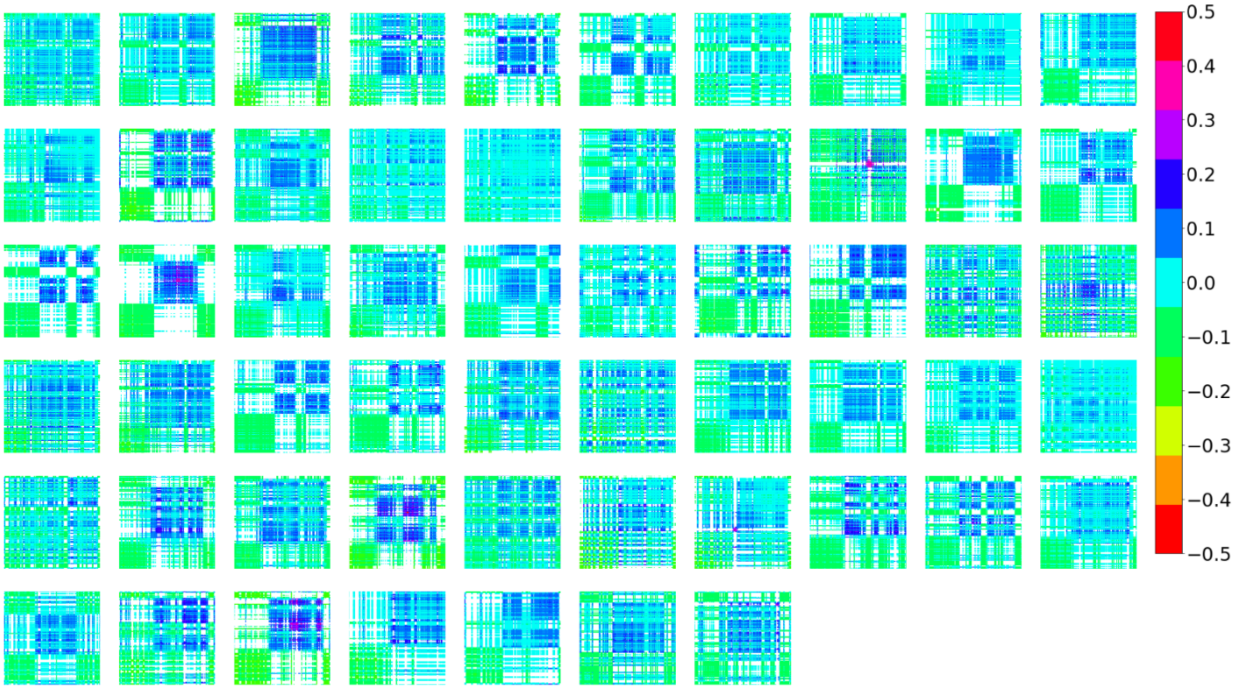

I. Children's CSI

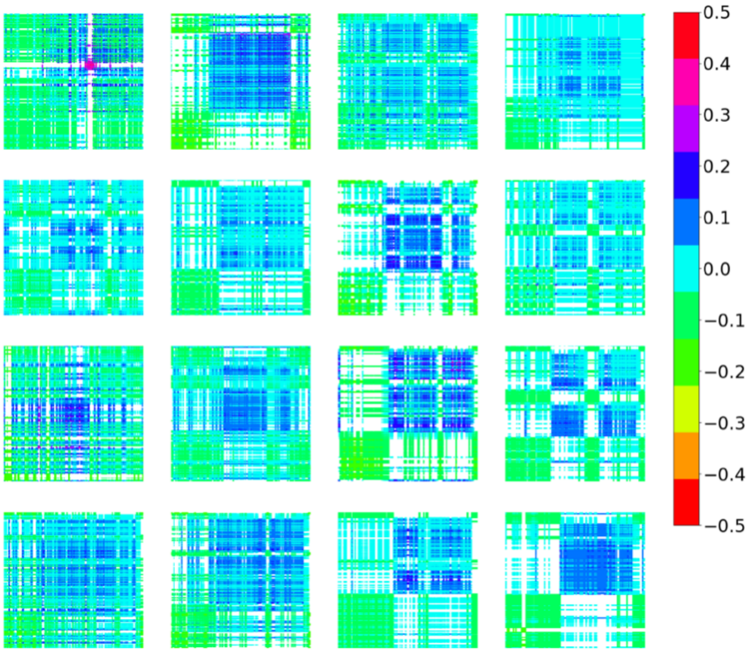

J. Children's CSI RPs SOM

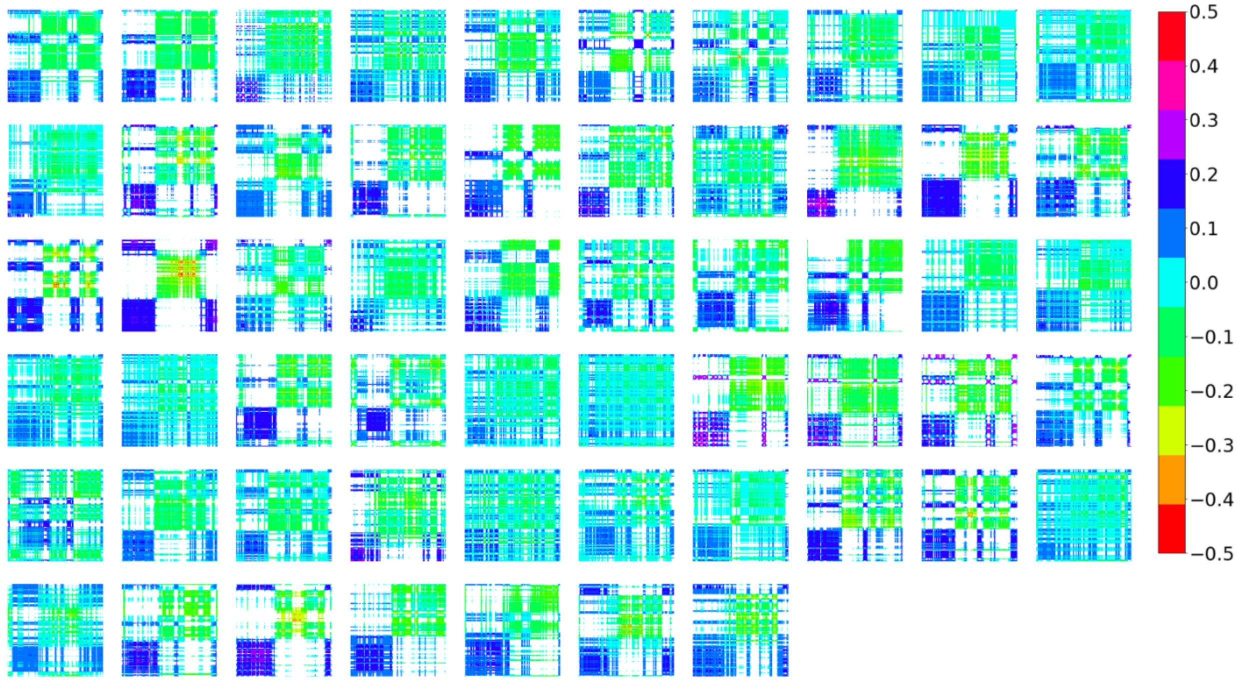

K. Children’s CVI

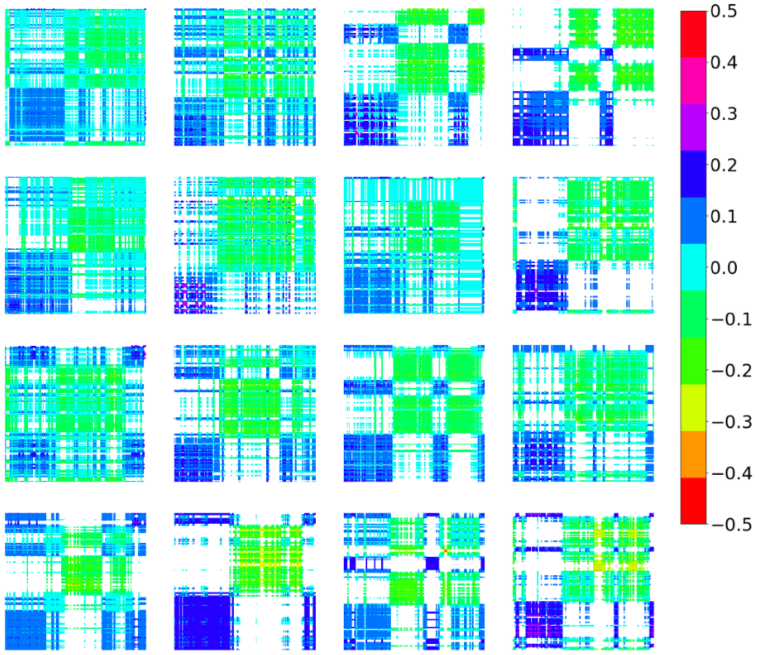

L. Children’s CVI RPs SOM
