# Supplementary material for: Mother-child autonomic nervous system interaction as an indication of parental stress: 24-hour cross recurrence plot analysis
Source: PLoS One. 2025 Sep 12;20(9):e0320175. doi: 10.1371/journal.pone.0320175 (PMC12431278; doi:10.1371/journal.pone.0320175)
Supplement: Attachment — Submitted filename: Response_letter.docx [file pone.0320175.s005.docx]

**Response letter**

**Response to the Reviewers:**

Dear Reviewers:

Thank you for your valuable feedback on our manuscript and providing us with heuristic suggestions. The following are the responses to each question and the corresponding revisions in the modified manuscript.

**Responses to Reviewer 1:**

*Authors made a review or critique of the study titled* *"Mother-child autonomic nervous system interaction as an indication of parental stress: 24-hour cross recurrence plot analysis.*

Dear reviewer, I guess this is a misunderstanding of our manuscript. The manuscript is not a review paper, but is a research paper titled "Mother-child autonomic nervous system interaction as an indication of parental stress: 24-hour cross recurrence plot analysis."

a. *What is the primary objective of the study, and how does it address gaps in existing research on mother-child physiological interactions?*

**Response**: The primary objective of this study was to explore the association between childcare stress and long-term ANS interactions between mothers and children. Existing research has neglected the analysis of prolonged daily interactions, particularly those involving circadian rhythms, and has often failed to account for the inherent nonlinear dynamics of ANS regulations. By employing the CRPs, which effectively capture these nonlinear interactions, this study directly addresses these gaps.

We have added clarifications of these objectives in the Introduction section, the 5^th^ paragraph, lines 84-89.

b. *How does this study differ from previous research that focused on short-term, activity-based interactions?*

**Response**: Unlike short-term interactions, the interactive patterns in such long-timescale signals, such as circadian rhythms, which offer critical insights into the body’s natural regulatory patterns, may also be closely related to caregiving stress.

We added content to emphasize the significance of circadian rhythms in capturing continuous stress-related physiological fluctuations over extended periods, differentiating our approach from previous short-duration studies, in the Introduction section at the end of the 2^nd^ paragraph, lines 55-57.

c. *What is the significance of using 24-hour at-home data for analyzing mother-child autonomic nervous system interactions?*

**Response**: Using 24-hour at-home data allows for the investigation of authentic physiological interactions within natural environments, capturing inherent circadian variability and stress resilience in long-term physiological interactions not observable in shorter or laboratory-based studies.

We clarified the scientific importance of realistic, ecological validity provided by 24-hour home data in the Introduction at the end of the 3^rd^ paragraph, lines 67-70.

d. *Why were cross-recurrence plots chosen as the method for quantifying interactive patterns, and how do they capture the nonlinear dynamics of autonomic nervous system signals?*

**Response**: CRPs were selected because they are specifically designed to visualize and quantify nonlinear interdependencies and synchronization between two physiological signals. The nonlinear dynamics refer to the complexity and irregularity in time series signals that result from the intricate feedback mechanisms regulating heart rate variability, which linear methods cannot adequately describe.

We explicitly detailed what nonlinear dynamics entail and how CRPs uniquely capture these complex interactions in the Related Techniques section in the 1^st^ paragraph, lines 214-218.

e. *What key findings emerged from the study regarding the relationship between mother-child autonomic nervous system patterns and maternal parenting stress?*

**Response**: The key finding is that CRPs provide a more effective framework than individual RPs for predicting maternal stress due to their ability to capture the interactive and relational nature of physiological regulation between mother and child. While the visual structure of the CRPs was not directly interpreted in this study (due to down-sampling via SOM), the statistical superiority of CRP-based models highlights the theoretical and empirical importance of dyadic physiological coupling in understanding parenting stress.

We specified these points in the Parenting stress predicted by the CRPs section, the 4^th^ paragraph, lines 400-407.

f. *How do the day-to-night autonomic nervous system patterns, quantified through cross-recurrence plots, improve the prediction of maternal parenting stress compared to individual autonomic nervous system activity analysis?*

**Response**: CRPs enable the quantification of interpersonal synchrony across time by visualizing cross-subject recurrence, which is lost in RPs. This quantification, through recurrence structure encoded into SOMs, allowed us to generate more robust and accurate predictions of maternal stress than analyzing individual ANS dynamics alone.

We have added an explanation about how CRPs specifically capture inter-subject recurrence, improving prediction over single-subject analyses in the Parenting stress predicted by the CRPs section in the 5^th^ paragraph, lines 408-421.

g. *What are the potential implications of these findings for understanding and addressing parental stress in real-world settings?*

**Response**: The current findings will allow the implementation of Public Health policies and programs aiming at fostering the mental health of parents and caretakers who are normally exposed to the additional stress of childcaring. Potential real-world implications include the development of wearable physiological monitoring systems to alert caregivers about stress risks and targeted therapeutic strategies to improve caregiver-child physiological synchronization.

We listed detailed practical implications, highlighting various applicable contexts in the Discussion section in the last paragraph, lines 481-486.

h. *How might this research contribute to the development of interventions or tools for parents experiencing childcare stress?*

**Response**: The findings of this research can guide the creation of specific biofeedback and personalized stress reduction programs that consider caregiver-child synchronization patterns.

We added illustrations about the detailed, concrete intervention strategies derived from this research in the Discussion section in the last paragraph, lines 486-488.

i. *What limitations might exist in using 24-hour at-home data and cross-recurrence plot analysis for this type of research?*

**Response**: Although this study showed that circadian rhythms—especially the interactive patterns between mother and child dyads—have a close relationship with mothers’ daily parenting stress, some limitations exist and need further exploration. For example, only physiological data were used to predict caregivers’ mental stress. Behavioral data such as the sleep/wake cycles, physical contact between interactive partners, and environment-sharing conditions are necessary to identify the reasons for the distinct interactive patterns of ANS activities. In addition, there is variability in the 24-hour at-home data quality due to participant adherence to wearable device protocols, potential disruptions in normal activities, and signal artifacts from natural movements. Such noises will influence the quantifications of CRPs.

We comprehensively detailed specific methodological limitations in the Discussion section, the 7^th^ paragraph, lines 472-475.

j. *What future research directions are suggested by the findings of this study, particularly in terms of exploring circadian rhythms and stress-related physiological interactions?*

**Response**: Future research will integrate comprehensive behavioral assessments alongside physiological data, explore long-term developmental trajectories of circadian synchronization, and evaluate interactions designed to enhance the parents’ caregiving life.

Explicit and detailed future research directions have been added in the Discussion section, the 7^th^ paragraph, lines 476-479.

k. *Are the concepts of "cross-recurrence plots" and "nonlinear dynamics of autonomic nervous system signals" clearly explained for readers unfamiliar with these methods?*

**Response**: More explanations on the technologies were added in the Cross recurrence plot section, the 3^rd^ paragraph, lines 268-271--CRPs visually quantify how two separate physiological signals simultaneously exhibit similar or synchronous nonlinear dynamics, reflecting complex physiological feedback loops that regulate the autonomic nervous system, which linear statistics fail to capture.

In addition, for the explanations on the RPs and CRPs, we used the sine signal as examples, which are easier for readers who are unfamiliar with the technologies to understand.

We also included the explanation of the technology from RP, then extended it to CRP, making it easier to understand by the readers.

l. *How well does the abstract convey the complexity of the interplay between maternal and child autonomic nervous system activities?*

**Response**: The following contents were added to the end of the abstract to illustrate clearly the complexity of the interplay between maternal and child autonomic nervous system activities: “The cross-recurrence plots effectively quantified complex, nonlinear, day-to-night interactions of mother-child autonomous nervous system activities and significantly predicted maternal parenting stress, offering novel insights into daily parental stress dynamics beyond recurrence plots that merely quantify the activities from individual systems.”

m. *How well does the abstract convey the complexity of the interplay between maternal and child autonomic nervous system activities?*

**Response**: This study offers several novel contributions to the field of parental stress and physiological synchrony:

First application of CRP to 24-hour ANS dyadic data in naturalistic caregiving settings: While previous studies have used CRPs in behavioral contexts or short-term physiological recordings, this is one of the first to apply CRPs to long-form, home-based ANS recordings (SDNN, CSI, CVI) of both mothers and children across 24 hours.

Integration of circadian rhythm and nonlinear synchrony: The study highlights how physiological synchrony—particularly across circadian cycles—relates to maternal stress. It contributes to the understanding of how long-timescale patterns (e.g., day-night transitions) impact co-regulation and stress.

Methodological innovation combining CRP and SOM: This is the first study to use a SOM-based representation of CRPs to predict psychological indices like the Parenting Stress Index (PSI), bridging nonlinear dynamics, unsupervised learning, and psychometric assessment.

Empirical evidence for dyadic over individual analysis: The study shows that mother-child physiological interactions are more predictive of parenting stress than individual ANS data. This finding has theoretical implications for how stress should be modeled—in relational, not individual, terms.

n. *How does the study advance our understanding of the role of circadian rhythms in stress-related physiological interactions?*

**Response**: This study advances understanding of the role of circadian rhythms in stress-related physiology in the following ways:

Linking circadian synchrony to parenting stress: The study demonstrates that day-to-night alignment in mother-child ANS signals (captured by CRPs) is significantly associated with maternal PSI scores. This extends prior research by showing that circadian alignment is not just a biological feature but a meaningful social and psychological variable.

Quantifying synchrony across circadian cycles: Unlike linear measures or individual-based RPs, CRPs allow a structured representation of how circadian phases in two individuals co-evolve, showing stronger and more interpretable links with stress outcomes.

Introducing a new lens to study stress resilience: By showing that synchronized circadian rhythms correlate with reduced stress, the study supports theories of dyadic allostasis, where shared rhythms serve a protective function in emotionally demanding contexts like caregiving.

Providing actionable metrics for future intervention: These findings open the possibility of using circadian synchrony as a marker in wearable or biofeedback systems for early identification of at-risk parenting scenarios.

**Responses to Reviewer 2:**

*I would like to appreciate the work of the author. The paper looks good both technically and in standard quality.*

**Response**: Thank you for your recognition of our work and your useful suggestions for revisions.

1. *Improve the quality of the image, as it looks blurred.*

**Response**: We have increased the resolution of the figures in the updated version to make it easier to visualize. Also, the figures with high resolution were attached to the submission as separated files.

*2. Most of the references look like older papers. Check to see if they are really related papers.*

**Response**: The following references are added to this manuscript:

2. Morgan J K, Santosa H, Conner K K, et al. Mother–child neural synchronization is time linked to mother–child positive affective state matching. Social Cognitive and Affective Neuroscience, 2023, 18(1): nsad001.

7. Capraz Y Z, Konrad K, Reindl V. Concurrent and lagged physiological synchrony during mother–child interaction and their relationship to positive affect in 8-to 10-year-old children. Scientific Reports, 2023, 13(1): 17744. https://doi.org/10.1038/s41598-023-43847-8

9. Matsunaga M, Takeuchi M, Watanabe S, et al. Intestinal microbiome and maternal mental health: Preventing parental stress and enhancing resilience in mothers. Communications Biology, 2024, 7(1): 235. 10.1038/s42003-024-05884-5

25. Drews H J, Felletti F, Kallestad H, et al. Using cross-recurrence quantification analysis to compute similarity measures for time series of unequal length with applications to sleep stage analysis. Scientific Reports, 2024, 14(1): 23142. https://doi.org/10.1038/s41598-024-73225-x
