# Supplementary material for: Mother-child autonomic nervous system interaction as an indication of parental stress: 24-hour cross recurrence plot analysis
Source: PLoS One. 2025 Sep 12;20(9):e0320175. doi: 10.1371/journal.pone.0320175 (PMC12431278; doi:10.1371/journal.pone.0320175)
Supplement: Attachment — Submitted filename: Reviewer Comments.docx [file pone.0320175.s002.docx]

Authors made a review or critique of the study titled **"Mother-child autonomic nervous system interaction as an indication of parental stress: 24-hour cross recurrence plot analysis.**

**These are the following research questions to answer by the authors**

a. What is the primary objective of the study, and how does it address gaps in existing research on mother-child physiological interactions?
b. How does this study differ from previous research that focused on short-term, activity-based interactions?
c. What is the significance of using 24-hour at-home data for analyzing mother-child autonomic nervous system interactions?
d. Why were cross-recurrence plots chosen as the method for quantifying interactive patterns, and how do they capture the nonlinear dynamics of autonomic nervous system signals?
e. What key findings emerged from the study regarding the relationship between mother-child autonomic nervous system patterns and maternal parenting stress?
f. How do the day-to-night autonomic nervous system patterns, quantified through cross-recurrence plots, improve the prediction of maternal parenting stress compared to individual autonomic nervous system activity analysis?
g. What are the potential implications of these findings for understanding and addressing parental stress in real-world settings?
h. How might this research contribute to the development of interventions or tools for parents experiencing childcare stress?
i. What limitations might exist in using 24-hour at-home data and cross-recurrence plot analysis for this type of research?
j. What future research directions are suggested by the findings of this study, particularly in terms of exploring circadian rhythms and stress-related physiological interactions?
k. Are the concepts of "cross-recurrence plots" and "nonlinear dynamics of autonomic nervous system signals" clearly explained for readers unfamiliar with these methods?
l. How well does the abstract convey the complexity of the interplay between maternal and child autonomic nervous system activities?
m. What makes this study novel or significant in the field of parental stress and physiological interactions?
n. How does the study advance our understanding of the role of circadian rhythms in stress-related physiological interactions?
